# Acute kidney injury after propofol or sevoflurane anaesthesia for colorectal cancer surgery: a secondary analysis

**DOI:** 10.1111/anae.70198

**Published:** 2026-03-10

**Authors:** Micael Taavo, Robert Frithiof, Mats Enlund, Stephanie Franzén

**Affiliations:** ^1^ Uppsala University Uppsala Sweden

Acute kidney injury is a frequent postoperative complication associated with substantial morbidity and mortality [1]. Although haemodynamic instability and surgical complexity are well‐established risk factors, the influence of anaesthetic maintenance technique on acute kidney injury incidence and severity remains poorly understood [1, 2, 3]. Experimental and clinical data suggest a plausible biological rationale for differential renal effects between drugs, including altered renal perfusion and sympathetic activation associated with volatile inhalational anaesthesia [4].

To evaluate whether the choice of anaesthetic drug influences postoperative acute kidney injury, we performed a secondary analysis of a multicentre randomised controlled trial [5]. Patients undergoing colorectal cancer surgery with available peri‐operative creatinine measurements were included. The primary outcome was postoperative acute kidney injury within 7 days, as defined by KDIGO creatinine criteria [6]. Logistic regression was used to assess the association between anaesthetic technique and acute kidney injury, adjusting for predefined intra‐operative variables reflecting haemodynamic and procedural stress (hypotension, blood loss, fluid balance and duration of anaesthesia). Sensitivity analyses using penalised regression models (ridge, lasso and elastic net) yielded consistent results.

Of 3256 patients assessed in the parent trial, 3229 had available peri‐operative creatinine measurements and were included in the acute kidney injury incidence analysis. There were then 3213 patients with complete data for all prespecified covariates included in the adjusted analyses (Table 1). There were 2769 (86%) patients classified as ASA physical status 1–2 and 249 (8%) of the overall cohort developed postoperative acute kidney injury. There was no significant difference in acute kidney injury incidence between patients receiving propofol‐ and sevoflurane‐based anaesthesia (121, 8% vs. 128, 8%, p = 0.700). The timing of acute kidney injury diagnosis within 7 postoperative days as assessed using Kaplan–Meier methods did not differ between groups.

**Table 1:** Baseline and intra‐operative characteristics of the regression analysis cohort by anaesthetic drug. Summarised data are based on the complete‐case regression cohort used for the adjusted analyses. Values are median (IQR [range]), number (proportion) or mean (SD).

	Total	Propofol	Sevoflurane
	n = 3213	n = 1603	n = 1610
Age; y	67 (58–74 [18–99])	67 (59–74 [20–99])	67 (58–74 [18–94])
Sex; female	1337 (42%)	653 (41%)	684 (43%)
BMI; kg.m^‐2^	24.8 (4.0)	24.8 (4.0)	24.8 (4.1)
ASA physical status 3 or 4	453 (14%)	235 (15%)	218 (14%)
Pre‐existing kidney disease	70 (2%)	38 (2%)	32 (2%)
Time MAP < 65 mmHg; min	0 (0–20 [0–395])	0 (0–20 [0–315])	0 (0–20 [0–395])
Blood loss; ml	50 (30–100 [0–4800])	50 (30–100 [0–4800])	50 (30–100 [0–4250])
Fluid balance; ml	1350 (850–1800 [‐1284–5550])	1350 (850–1800 [‐1284–5550])	1338 (800–1800 [‐800–4661])
Duration of anaesthesia; min	250 (202–329 [60–1418])	253 (206–327 [93–1418])	250 (198–330 [60–1220])

**Figure 1:**
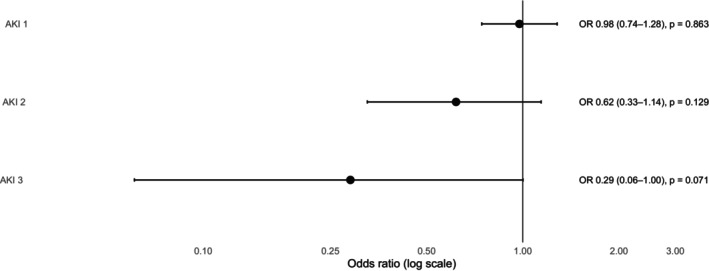
Association between anaesthetic drug and postoperative acute kidney injury (AKI). Adjusted ORs with 95% confidence intervals for postoperative acute kidney injury by KDIGO stage (1–3), comparing sevoflurane‐ with propofol‐based anaesthesia. Estimates are derived from multivariable logistic regression models adjusted for time with mean arterial pressure < 65 mmHg, blood loss, fluid balance and duration of anaesthesia. Vertical line indicates an OR of 1.

Adjusted analyses showed no association between anaesthetic drug and acute kidney injury for any KDIGO stage (Fig. 1). For acute kidney injury stages 2 and 3, event numbers were low, resulting in wide confidence intervals compatible with both increased and decreased risk. In this cohort, the precision of the estimates allows exclusion of absolute between‐group differences in postoperative acute kidney injury of ≥ 2.8 percentage points in absolute risk, although smaller differences cannot be ruled out.

This secondary analysis of a large, international randomised trial of anaesthetic drugs found no evidence of a clinically meaningful difference in postoperative acute kidney injury between propofol‐ and sevoflurane‐based anaesthesia in patients undergoing laparoscopic colorectal cancer surgery. The precision of the estimates allows exclusion of moderate differences in overall acute kidney injury risk, particularly for stage 1 acute kidney injury. Because allocation to anaesthetic drug was randomised in the parent trial, confounding by indication was minimised thus strengthening causal inference regarding agent‐specific renal effects.

From a clinical perspective, these findings suggest that the choice of anaesthetic maintenance technique alone should not be expected to influence postoperative acute kidney injury risk in this setting, supporting equipoise between propofol and sevoflurane for renal outcomes in low‐risk colorectal surgery and allowing anaesthetic choice to be guided by other clinical priorities.

These results should be interpreted in the context of previous studies reporting inconsistent findings when comparing volatile and intravenous anaesthesia. Experimental and physiological data provide a plausible biological rationale for differential renal effects between agents, including altered renal perfusion and sympathetic activation under volatile anaesthesia [4, 7, 8]. However, clinical studies in gastrointestinal surgery have yielded mixed results [2, 3], and our findings support the notion that such mechanistic differences do not necessarily translate into clinically detectable acute injury in well‐optimised, low‐risk patients.

The absence of an observed difference may be influenced by the characteristics of the study population. Most patients (86%) were ASA physical status 1–2 and underwent laparoscopic colorectal surgery, a setting characterised by relatively limited surgical trauma and blood loss. In such contexts, a possible difference in renal blood regulation between anaesthetic drugs may be insufficient to produce measurable creatinine‐based acute kidney injury.

Our study also has limitations. Acute kidney injury was defined using plasma creatinine alone, which may underestimate mild events, and the analysis was not designed to assess high‐risk subgroups or severe acute kidney injury. Accordingly, potential differences between anaesthetic drugs may still be clinically relevant in patients with impaired renal reserve, greater haemodynamic instability or higher surgical risk. These populations warrant targeted investigation in future studies.
